# Demographic Variations of MERS-CoV Infection among Suspected and Confirmed Cases: An Epidemiological Analysis of Laboratory-Based Data from Riyadh Regional Laboratory

**DOI:** 10.1155/2020/9629747

**Published:** 2020-02-19

**Authors:** Asmaa Altamimi, Raghib Abu-Saris, Ashraf El-Metwally, Taghreed Alaifan, Aref Alamri

**Affiliations:** ^1^College of Public Health and Health Informatics, King Saud Bin Abdulaziz University for Health Sciences, Riyadh, Saudi Arabia; ^2^National Health Laboratory, Saudi Center for Disease Prevention and Control (Saudi CDC), Riyadh, Saudi Arabia; ^3^King Abdullah International Medical Research Center (KAIMARC), Riyadh, Saudi Arabia; ^4^Department of Virology and Cytogenetics, Riyadh Regional Laboratory, Riyadh, Saudi Arabia

## Abstract

*Introduction*. Middle East respiratory syndrome coronavirus was first recognized in September 2012 in Saudi Arabia. The clinical presentations of MERS and non-MERS SARI are often similar. Therefore, the identification of suspected cases that may have higher chances of being diagnosed as cases of MERS-CoV is essential. However, the real challenge is to flag these patients through some demographic markers. The nature of these markers has not previously been investigated in Saudi Arabia, and hence, this study aims to identify them.

**Methods:**

It was a surveillance system-based study, for which data from a total of 23,646 suspected patients in Riyadh and Al Qassim regions were analyzed from January 2017 until December 2017 to estimate the prevalence of MERS-CoV among suspected cases and to determine potential demographic risk factors related to the confirmation of the diagnosis.

**Results:**

Of 23,646 suspected cases, 119 (0.5%) were confirmed by laboratory results. These confirmed cases (67.2% of which were males) had a mean age of 43.23 years (SD ± 22.8). Around 42.2% of the confirmed cases were aged between 41 and 60 years and about 47% of confirmed cases had their suspected specimen tested in the summer. The study identified three significant and independent predictors for confirmation of the disease: an age between 41 and 60 years, male gender, and summer season admission.

**Conclusion:**

The study provides evidence that the MERS-CoV epidemic in the subject regions has specific characteristics that might help future plans for the prevention and management of such a contagious disease. Future studies should aim to confirm such findings in other regions of Saudi Arabia as well and explore potential preventable risk factors.

## 1. Introduction

A respiratory viral disease caused by the Middle East Respiratory Syndrome CoronaVirus (MERS-CoV) was first isolated in 2012, in a 60-year-old man who died in Jeddah, KSA due to severe acute pneumonia and multiple organ failure [1]. Since then, 27 countries have reported the presence of this virus, including the 12 countries of the Eastern Mediterranean region. Several outbreaks have occurred in multiple countries including Saudi Arabia, the United Arab Emirates and the Republic of Korea [2]. Recent data from WHO shows that almost 80% of the reported MERS-CoV cases have been in Saudi Arabia; a total of 1,983 cases, with 745 related deaths and a case-fatality rate of 37.5 [3].

Travel-related cases have occurred in countries like Greece, Germany, France, Philippines, the United Kingdom, Malaysia, Egypt, Italy, the Netherlands, Turkey, South Korea, Algeria, Austria, China, and the United States [4]. To date, the largest outbreak of MERS-CoV, after Saudi Arabia, occurred in South Korea during the period between May and July of 2015, with 186 reported cases, 39 deaths, and a case-fatality rate (CFR) of 21% [5, 6]. Very limited evidence is available for exploring the epidemiology of this virus among the pediatric population [7]. The literature shows that MERS-CoV infects males more than females [8, 9]. The case-fatality rate of men (52%) is higher than that of women (23%) [10]. Males with a history of serious medical conditions are highly susceptible to this infection. Moreover, the mean age of infection in adults is 60 years [10].

The mode of transmission is not entirely understood yet [2]; however, human-to-human [11] and zoonotic sources of transmission [12] have been documented in many studies. Dromedary camels are the major animal source of MERS-CoV transmission to humans. Interhuman transmission of the virus did not occur easily, but it is seen mainly in patients' families and healthcare settings [2]. Clinical pictures of this infection varied from asymptomatic to mild respiratory symptoms to severe respiratory distress and death [2]. Severe ailment can often cause respiratory catastrophes that need mechanical ventilation and support in ICUs across different healthcare settings [4]. Studies have suggested an incubation period of 16 days with a mean of 5-6 days [12, 13], while the median time until death is 11–13 days (range 5–27 days) among severely ill patients [13]. The gold standard test for the detection of this virus is real-time reverse-transcription polymerase chain reaction (rRT-PCR) assays [14].

There is no specific treatment for MERS-CoV. Like most viral infections, the treatment options are supportive and symptomatic [2]. At present, no vaccine exists for preventing the infections of MERS-CoV. The CDC indicated that preventative actions should be taken for any type of respiratory illness [4]. Such actions include washing hands with water and soap for around 20 seconds or using hand sanitizers with alcohol if no water is available. One must cover their nose and mouth during instances of sneezing and coughing with a tissue and avoid touching the mouth, nose, or eyes with their hands until washed properly. Repeatedly touched surfaces, such as door knobs, should be disinfected and cleaned regularly. Intimate personal contact, e.g., kissing, and sharing cups or eating utensils must also be avoided [15].

Many studies have been conducted in recent years in Saudi Arabia to combat this deadly disease. A large multicentre study showed that it is nearly impossible to differentiate between patients of MERS-CoV and non-MERS-CoV just on the basis of clinical presentation [16]. Another cohort study, which was hospital-based (17 cases vs. 82 controls), found that there were statistically significant differences in terms of gender, clinical, and radiographic presentations [17]. Similarly, two more single-centre case control studies reported that the presenting symptoms of MERS-CoV infection were not specific [18, 19].

Physicians and public health practitioners need to identify suspected cases which have higher chances of diagnosis as confirmed cases prior to laboratory testing (which usually takes between 12 and 24 hours). Identification of a confirmed case is necessary to implement preventive strategies to combat the spread of the disease to family members and hospital healthcare workers [20]. Mild symptomatic cases, which result in a positive PCR, may be isolated at home. Severe to moderate cases should be admitted to and isolated in a hospital until they improve and then be discharged for isolation at home for an extended period. Both mild and severe cases are retested after 7 days, and the test is subsequently repeated after every 3 days until a negative result is obtained [20].

Identifying suspected cases which may have higher chances of getting diagnosed as a confirmed case and implementing strict procedures on them might offer the best solution. The challenge is to flag these patients by some demographic markers, as the clinical presentation of MERS-CoV infected patients were non-specific. Therefore, we aimed to identify some demographic markers specific to confirmed cases of MERS-CoV. The nature of these markers has not been investigated in Saudi Arabia, and hence this study aims to identify them.

## 2. Materials and Methods

### 2.1. Study Design and Setting

A cross-sectional study was conducted at the regional laboratory and blood bank, located at Shumaisi Hospital in Riyadh, KSA. The laboratory has received the Central Blood Banks and Reference Laboratories Accreditation Program Saudi Central Board for Accreditation of Healthcare Institution (CBAHI) 2018 [21].

### 2.2. Sampling Technique

Data were collected during the period of January 2017 to December 2017. All patients in Riyadh and Al-Qassim regions who had their samples tested at Riyadh regional lab during the study period were considered as suspected cases. The study had two aims: descriptive and analytical. For the descriptive aim, we estimated the prevalence of MERS-CoV. For the analytical aim, a binary logistic regression model was developed. In this model, we included the risk factors of gender, age, seasons, nationality, healthcare status (yes/no), hospitals, and area of residence. Data were cross-checked with a lab-computerized database. Further data were collected on demographic characteristics (age and sex), underlying nationality, and health care status.

### 2.3. Statistical Analyses

We collected data from 25,400 cases, of which 23,646 suspected cases of MERS-CoV were included in the final analysis. Data were cleaned, entered, stored, and managed with an excel database and IBM SPSS Version 25. The statistical analyses consisted of descriptive counts and percentages. For those continuously scaled items, nonparametric statistics (medians, interquartile ranges, minimum, and maximum) were used to describe the distribution. A logistic regression analysis was used to identify predictors of confirmation of infection within the suspected cases groups. At first, univariate analyses were conducted to estimate the unadjusted contribution and to determine the significant risk factors. This was followed by a multivariate logistic regression analysis to estimate the independent contribution of each covariate. To determine significant factors, a *p* value below 0.05 and a 95% confidence interval were considered.

### 2.4. Ethical Approval

Full ethical approval was obtained by the Ministry of Health Institutional Review Board.

### 2.5. Operational Definition

#### 2.5.1. Suspected Case

According to MERS-CoV guideline version 5, a MERS-CoV suspected case is defined based on the age group, clinical presentation, and epidemiologic links (Table 1) [20].

#### 2.5.2. Confirmed Case

A confirmed case is defined as a suspected case with laboratory confirmation of MERS-CoV infection [20].

#### 2.5.3. MERS-CoV Gold Standard Test

Real-time reverse-transcription polymerase chain reaction (rRT-PCR) assays were performed. Laboratory confirmation of MERS-CoV infection requires a positive rRT-PCR result for at least two specific genomic targets: region upstream and open reading frame1a (upE and ORF1a) [20–22].

## 3. Results

### 3.1. Demographics of Suspected Cases

A total of 23,646 of MERS-CoV suspected cases were included in this study, of which 52.3% were males (*n* = 12376) and 47.7% were females (*n* = 11270). The age of individuals with suspected cases ranged between 0 to 92 years with a mean age of 43.23 and a SD of ±22.83 years. Younger patients (age group 20–40 years) were mostly presented as suspected cases (*n* = 8921, 37.7%). The prevailing season, during which highest percentage of admission of suspected cases (*n* = 7233, 30.6%) was reported, was Fall. Only a minority of suspected cases were confirmed as having detected MERS-CoV (0.5%, 95% CI 0.42–0.60) Table 2.

### 3.2. Demographics of Confirmed Cases

Of 119 confirmed cases of the MERS-CoV, the male-female ratio was approximately 2 : 1 (67% male and 33% female). The highest prevalence of MERS-CoV infected cases (41.2%) was seen in the age group 41–60 years, and most of the confirmed cases were recorded during summer. The majority consisted of non-healthcare workers (74.8%), and most were Saudi nationals (54.6%). Around 25.2% of such cases eventually died due to MERS-CoV.  Table 3 shows the sample characteristics of confirmed cases.

Logistic regression analysis showed that the adjusted odds of MERS-CoV in males increased twofold as compared with females (A.OR: 1.88, 95% CI: 1.28–2.765, *P* value = 0.001). The adjusted odds of MERS-CoV remained significant among different age groups; the odds of patients aged between 20–40 years increased threefold (A.OR: 3.11, 95% CI: 1.104–8.76, *P* value = 0.032), whereas in the age group of 41–60 years, it increased further to a risk that was six times higher (A.OR: 6.01, 95% CI: 2.034–16.83, *P* value = 0.001). In the age category above 61 years, it increased threefold (A.OR: 3.046, 95% CI: 1.053–8.12, *P* value = 0.040) as compared with the 0–19 years old group. The risk of MERS-CoV was significantly higher during the summer (A.OR: 3.3, 95% CI: 1.83–6.19, *P* value < 0.001) and spring seasons (A.OR: 2.125, 95% CI: 1.094–4.129, *P* value = 0.026) as compared with that in the winter in the multivariate analysis (Table 4).

## 4. Discussion

This cross-sectional study about the epidemiological analysis of MERS-CoV infection laboratory-based data was conducted in Riyadh over a one-year period (2017). A total of 23,646 suspected cases were included in the results. Of the total suspected cases, 119 cases had been confirmed via laboratory results. All the confirmed cases are reported to MOH through HESN (health electronic surveillance networks) and to the World Health Organization (WHO) through the International Health Regulations (IHR), National Focal Point of Saudi Arabia. We found that MERS-CoV infection was found significantly in people aged between 41 and 60 years and was reported most commonly during the summer season. The odds of infection among males were found to be twice as high as that of females with suspected cases.

During the study period, i.e., the year 2017, only 119 confirmed cases were reported, which means that the number of MERS-CoV infection cases has decreased in Riyadh and Al-Qassim regions in comparison to that of the last three years. From 2015 to 2016, there was a 25.4% decrease, whereas from 2016 to 2017, it decreased by 48.7%, which translates into a 50% decrease between the two periods. This also complements the findings reported by of Da'ar and Ahmed in their paper [23]. The predominance of infection in males was also observed in another study pwefromed in KSA (2015), which reported the percentage of confirmed cases among males to be 66%, compared with 34% among females [24].

It is worth mentioning that Saudi Arabia defines age categories differently from the WHO (children: 0–14, adult: otherwise) [20]. However, unlike the classification used in Saudi Arabia, we have followed the WHO categorization of age to differentiate between children/adolescents (0 to 19 years) and adults (20 years and older) as indicated in WHO reports for age-standardized population and in infectious diseases [25]. This categorization was also followed by Aly and his collaborators in their recent paper published in 2017 [14]. Adults were further subcategorized into three groups according to the age distribution of the study population using the following two cutoff points (age of 41 and age of 60) [14].

These data agreed with a previous surveillance study, which stated that the majority of confirmed cases of MERS-CoV were reported among people aged 40 and above [24]. In 2016, only 9 of 552 cases (1.6%) of MERS-CoV infection were found among pediatric patients. Moreover, the study which was conducted in King Fahad Medical City in Riyadh (KFMC) between January 2012 and December 2013 did not report any MERS-CoV cases among children [26]. The study which was conducted across the Gulf countries for four years by Mahmoud Aly et al. between 2012 and 2016 suggests that the prevalence and distribution of MERS-CoV were the highest-risk in elderly aged 60 years or above [14]. Similar to our results, this study also reported the highest number of confirmed cases during the summer season [14].

Among confirmed cases, only 25.2% were healthcare workers, whereas around 75% were non-healthcare workers. This is in agreement with the study done by Ahmad to estimate the survival rate in MERS-CoV globally prior to 26 January 2017; 86.9% were not health-care workers compared with 13.1% confirmed cases of healthcare workers [27]. Similarly, other studies also reported a lower prevalence in healthcare workers [28–30].

Our data reported a higher prevalence of infection among Saudi nationals as compared with non-Saudi. Another study also showed similar results but with a much higher percentage among Saudis, which may be due to the fact that it included Saudis from all regions [29]. There is no finding basis for comparison as such, because our study was focused on the Riyadh and Al Qassim regions only.

In our study, we detected a low prevalence (0.5%). The low positive predictive value of our lab results is not related to the low sensitivity and specificity of the lab assay. The estimated analytical sensitivity and specificity of the Real Star kit from Altona was reported to be 100% with no cross reactivity with other respiratory pathogens [31]. Moreover, this low predictive value in the lab results is related to the high burden of false positive cases referred to the lab. In fact, this research is just the starting point to shed the light on more factors that might help in putting more descriptive criteria to lower the financial and human resources burden.

To the best of our knowledge, no one has developed a logistic regression that focuses on demographic risk factors such as sex, age, and seasons prior to our study. However, it is worth mentioning that Ahmed et al. developed a risk prediction model that encompasses risk factors such as chest pain, leukopenia, and elevated aspartate aminotransferase (AST) [21]. However, further investigations are needed to confirm our findings.

One of the major strengths of our study is that it is a comprehensive regional study which included all the suspected cases of MERS-CoV in the Riyadh and Al-Qassim regions. Secondly, the external validity of our study is also expected to be high, as it covers the two regions completely, meaning that the records of all suspected cases in these two main regions in Saudi Arabia were included. Thirdly, the quality of the data is considered to be high, given that the contagious and life-threatening nature of this disease has led to strict obedience to rules which are enforced in a timely manner, thus ensuring accurate reporting of suspected cases. In addition to this, quality assurance policies are implemented at HESN in order to maintain the highest level of validity and reliability of the data collection process. The variables available for suspected cases were limited to demographics, which limited the scope of our research, but they provided valuable information to form a basis for future studies of a broader scope. Variables such as primary/secondary infections are vital pieces of information, but due the limitation of the data available, we could not determine their effects.

According to our knowledge, this is one of the few studies that have specifically investigated MERS-CoV risk factors in the Riyadh and Al-Qassim areas (two major regions in KSA). Given that all suspected and confirmed cases were included in this study, we assume that our results are generalizable for both the regions with confidence. It must be noted that the comparative group of this study is different from that of the previous ones, as we compared those with confirmed MERS-CoV with those with suspected MERS-CoV who have passed all stages of screening at the hospital, whereas other studies were hospital but not lab-based with an aim of identifying factors that help in suspecting rather than confirming cases. This might be the reason why we have found some significant demographic factors unlike other reports.

## 5. Conclusion

In conclusion, this research is about predictors for the confirmation of diagnosis among suspected cases only, meaning that the factors we found can help in identifying suspected cases that may have a higher chance of testing positive. This will help primary healthcare professionals to develop a better screening tool for suspected cases, as currently only a small minority of suspected cases are confirmed positive via lab results, consequently resulting in a lot of resources being spent to test thousands of samples, just for the identification of a few cases. The three factors we identified are important because, for example, a female, aged 18, presenting in winter will be less likely to be diagnosed than a male, aged 45, presenting in the summer, or, to give another example, a 60-year-old male who is presenting MERS-CoV signs with a negative lab result may need retesting.

Our study covered two main regions in Saudi Arabia and provides evidence that the MERS-CoV epidemic in these two regions has specific characteristics that might help future plans for prevention and management of such contagious diseases. Our results showed that only a minority of suspected cases are actually diagnosed with the disease, meaning that the procedures being implemented seemed to be highly sensitive but not highly specific. The majority of confirmed cases were male, aged 41 to 60 years, and presented to healthcare facilities in the summer. Future studies should aim to confirm such findings in other regions in Saudi Arabia, to explore potential preventable risk factors and go deeper to know the underlying factors that make male aged 41–60 more susceptible than others.

## Figures and Tables

**Table 1:** Definition of MERS-CoV suspected cases.

Age	Clinical presentation	Epidemiologic links
Adults	(i) Severe pneumonia (severity score ≥3 points) or ARDS (built on radiological or clinical evidence) (ii) Unexplained deterioration of a chronic condition of patients with congestive heart failure or chronic kidney disease on hemodialysis	Not required
Children and adult	(iii) Acute febrile illness (*T* ≥ 380 C) with/without respiratory symptoms or (iv) Gastrointestinal symptoms (diarrhea or vomiting) and leukopenia (WBC ≤ 3.5 × 109/L) or thrombocytopenia (platelets < 150 × 109/L)	Within 14 days before symptom onset: (1) Exposure to a confirmed case of MERS-CoV infection or (2) Visit to a healthcare facility where MERS-CoV patients(s) has recently (within 2 weeks) been identified/treated or (3) Contact with dromedary camels or consumption of camel products (e.g., raw meat, unpasteurized milk, urine)

**Table 2:** Prevalence of confirmed cases.

		*N*	%	95% CI
Suspected	Not confirmed	23,034	99.5	0.42–0.60
	Confirmed	119	0.5	
Total valid data		23,153	100	

**Table 3:** Sample characteristics of confirmed cases.

		*N*	%
Sex	F	39	32.8
	M	80	67.2
Age group	0–19 y	4	3.4
	20–40 y	41	34.5
	41–60 y	49	41.2
	>60 y	25	21.0
Season	Winter	13	10.9
	Spring	27	22.7
	Summer	56	47.1
	Fall	23	19.3
Healthcare worker	No	89	74.8
	Yes	30	25.2
Nationality	Saudi	65	54.6
	Non-Saudi	54	45.4
Death	Alive	89	74.8
	Deceased	30	25.2

**Table 4:** Logistic regression model of MERS-CoV among suspected and confirmed cases.

	Reference	Univariate				Multivariate			
		*P* value	OR	95% CI for OR		*P* value	OR	95% CI for OR	
				Lower	Upper			Lower	Upper
Male	Female	0.001	1.875	1.278	2.752	0.001	1.882	1.280	2.765
Age 20–40 y	Age 0–19 y	0.013	3.684	1.319	10.294	0.036	3.026	1.075	8.516
Age 41–60 y	Age 0–19 y	0.001	6.992	2.521	19.394	0.001	6.015	2.160	16.755
Age >60	Age 0–19 y	0.023	3.398	1.182	9.772	0.040	3.034	1.050	8.764
Spring	Winter	0.025	2.135	1.100	4.143	0.026	2.125	1.094	4.129
Summer	Winter	0.001	3.328	1.818	6.092	0.001	3.374	1.838	6.192
Fall	Winter	0.558	1.226	0.620	2.422	0.424	1.321	0.667	2.613

*α* = 0.05.

## Data Availability

The laboratory data used to support the findings of this study were provided by Riyadh Regional Laboratory under license and are not freely available. However, access to data will be considered from the corresponding author upon request.
